# IS ME IMPORTANT TO US? ACTOR AND PARTNER EFFECTS OF SELF-ACCEPTANCE ON SPOUSAL SUPPORT

**DOI:** 10.1093/geroni/igz038.2483

**Published:** 2019-11-08

**Authors:** Kyuho Lee

**Affiliations:** Daegu University, Gyeongsan, Korea, Republic of

## Abstract

Marital relationship is influenced by substantial and perceived support exchange. Therefore, it is important to find predictors of support-giving and support-receiving. However, very few studies attempted to do so. As some previous studies reported, individuals’ personality-related characteristics seem to play a significant role in the support exchange within couples. This study aimed to assess the effect of individuals’ self-acceptance on support exchange within older couples. Data of 2,082 heterosexual older couples aged between 50 and 85 in 2006 from the Health and Retirement Study were assessed utilizing an actor-partner inter-dependence model and growth curve model. For the actor effects, both husbands’ and wives’ higher levels of self-acceptance predicted their own perception of the support from a spouse. For the partner effects as well, individuals’ self-acceptance positively predicted their spouse’s perception of the received support. Husbands’ self-acceptance, however, was associated with a decrease in wives’ perceived support. We discuss the possible mechanism with regard to older adults’ self-acceptance and marital support. The findings of the current study contribute to the theories of spousal support and personality as well as in the practical settings of couple therapy and education.

